# Malaria control and prevention towards elimination: data from an eleven-year surveillance in Shandong Province, China

**DOI:** 10.1186/s12936-017-1708-0

**Published:** 2017-01-31

**Authors:** Xiangli Kong, Xin Liu, Hong Tu, Yan Xu, Jianbing Niu, Yongbin Wang, Changlei Zhao, Jingxuan Kou, Jun Feng

**Affiliations:** 1 Shandong Institute of Parasitic Diseases, Shandong Academy of Medical Sciences, No. 11 Taibai Zhong Road, Jining, 272033 Shandong China; 2 Jining No. 1 People’s Hospital, No. 6 Jiankang Road, Jining, 272011 Shandong China; 3 National Institute of Parasitic Diseases, Chinese Center for Disease Control and Prevention, Key Laboratory of Parasite and Vector Biology, Ministry of Health, WHO Collaborating Centre for Tropical Diseases, National Center for International Research on Tropical Diseases, No. 207 Ruijin Er Road, Shanghai, 200025 China

## Abstract

**Background:**

Shandong Province experienced a declining malaria trend of local-acquired transmission, but the increasing imported malaria remains a challenge. Therefore, understanding the epidemiological characteristics of malaria and the control and elimination strategy and interventions is needed for better planning to achieve the overall elimination goal in Shandong Province.

**Methods:**

A retrospective study was conducted and all individual cases from a web-based reporting system were reviewed and analysed to explore malaria-endemic characteristics in Shandong from 2005 to 2015. Annual malaria incidence reported in 2005–2015 were geo-coded and matched to the county-level. Spatial cluster analysis was performed to evaluate any identified spatial disease clusters for statistical significance. The space–time cluster was detected with high rates through the retrospective space–time analysis scanning using the discrete Poisson model.

**Results:**

The overall malaria incidence decreased to a low level during 2005–2015. In total, 1564 confirmed malaria cases were reported, 27.1% of which (n = 424) were indigenous cases. Most of the indigenous case (n = 339, 80.0%) occurred from June to October. However, the number and scale of imported cases have been increased but no significant difference was observed during months. Shandong is endemic for both *Plasmodium vivax* (n = 730) and *Plasmodium falciparum* (n = 674). The disease is mainly distributed in Southern (n = 710) and Eastern region (n = 424) of Shandong, such as Jinning (n = 214 [13.7%]), Weihai (n = 151 [9.7%]), and Yantai (n = 107 [6.8%]). Furthermore, the spatial cluster analysis of malaria cases from 2005 to 2015 indicated that the diseased was not randomly distributed. For indigenous cases, a total of 15 and 2 high-risk counties were determined from 2005 to 2009 (control phase) and from 2010 to 2015 (elimination phase), respectively. For imported cases, a total of 26 and 29 high-risk counties were determined from 2005 to 2009 (control phase) and from 2010 to 2015 (elimination phase), respectively. The method of spatial scan statistics identified different 13 significant spatial clusters between 2005 and 2015. The space–time clustering analysis determined that the most likely cluster included 14 and 19 counties for indigenous and imported, respectively.

**Conclusions:**

In order to cope with the requirements of malaria elimination phase, the surveillance system should be strengthened particularity on the frequent migration regions as well as the effective multisectoral cooperation and coordination mechanisms. Specific response packages should be tailored among different types of cities and capacity building should also be improved mainly focus on the emergence response and case management. Fund guarantees for scientific research should be maintained both during the elimination and post-elimination phase to consolidate the achievements of malaria elimination.

## Background

**Fig. 1:**
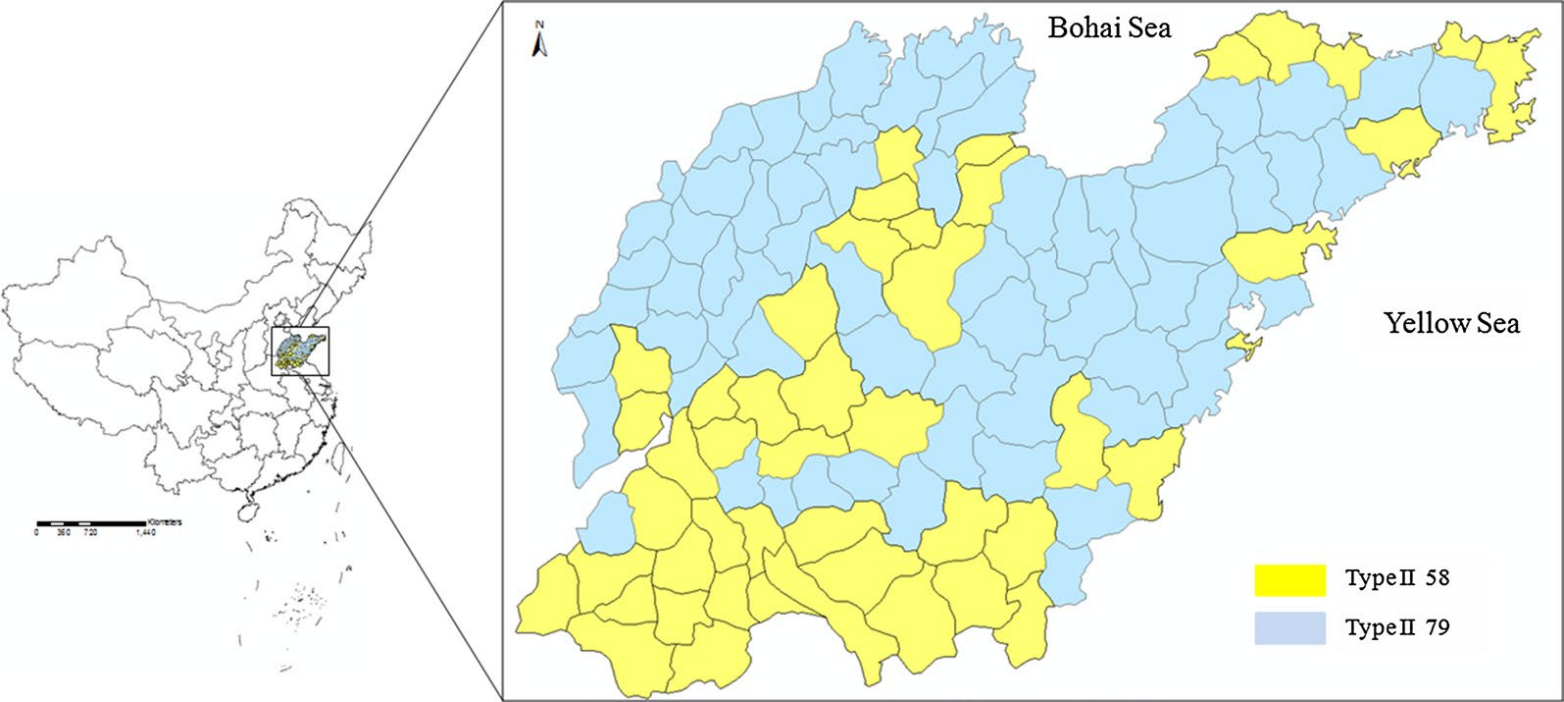
Location of Shandong Province in China. All Type II and Type III counties are labelled on the right

Shandong Province had been once a high malaria transmission area in China. The malaria annual morbidity reached the primary peak in 1961 recorded as 806.2 per 10,000, as well as a secondary peak in 1971 with 493.2 per 10,000, respectively [1]. After that, the disease burden was sharply reduced mainly due to the large-scale surveys and anti-malarial campaigns conducted on the areas with high transmission of malaria, where was prevalent in the southern and central region of Shandong. Recently, annual numbers of reported malaria cases have declined to less than 50 in 2000. From 2004 to 2008, the central region including Anhui, Jiangsu and Shandong provinces suffered the reemergence of local transmission and the incidence in Shandong was reached to peak in 2007 with 0.019 per 10,000 [2]. The epidemics had been well controlled after substantial efforts included the implementation of the National Malaria Control Programme (NMCP, 2006–2015) and National Malaria Elimination Action Plan (NMEAP, 2010–2020) [3, 4]. In addition, Shandong profited from the malaria control project supported by the Global Fund Fighting against AIDS, Tuberculosis and Malaria (GFATM), which contributed to decrease of malaria incidence significantly in China from 2002 to 2012. The NMEAP was launched in 2010 and all the counties were classified into four types (Types I to IV), and Shandong counties were considered to be either Type II or Type III (Fig. 1). Type II means that the local infections are detected in three consecutive years, and the incident rate is lower than 1/10,000 at least in one of those 3 years, and preventing any possible malaria cases and active foci is the main strategy to interrupt local transmissions. Type III implies that no local infections reported in 3 years, the capabilities of malaria surveillance and response will be emphasized to prevent malaria reintroduction (Table 1) [5]. Therefore, the malaria surveillance-response system in Shandong had reshaped in line with the guidelines to rapidly detect and identify all malaria infections and ensured appropriate treatment before any secondary cases or local transmission may occur.

**Table 1:** Stratification of malaria counties in relation to malaria transmission risk by NMEAP in China and Shandong Province

Type of county	Classification	No. (China/Shandong^a^)	Target (10,000) (China/Shandong^a^)
Type I	Local infections are detected in 3 consecutive years and the incident rate equals or higher than 1/10,000	75/0	3965/0
Type II	Local infections are detected in 3 consecutive years, and the incident rate is lower than 1/10,000 at least in one of those 3 years	687/58	44,792/4279
Type III	No local infections reported in 3 years	1432/79	62,281/4808
Type IV	Non-malaria-epidemic-area	664/0	18,882/0

^a^This indicated that the number of counties and target population in China and Shandong, respectively

Currently, the reported malaria incidence have decreased to low level and imported malaria have become the greatest challenge in Shandong. Since little research has been conducted on the transit period of malaria epidemiologic features from the control phase (2005–2009) to the elimination phase (2010–2015) in Shandong, the malaria epidemiologic characteristics in Shandong from 2005 to 2015 was investigated and analysed, so as to help adjusting and tailoring the elimination strategy and give some references to other provinces or countries with the similar elimination progress.

## Methods

### Study design

In China, malaria was one of the 37 notifiable infectious diseases and all epidemiologic information should be gathered and reported in the web-based reporting system launched in 2004. China’s information system for infectious system vertically covers all medical institutions at the national, provincial, prefectural and county levels. The system also horizontally covers CDCs and public health administration departments in all regions. In this study, malaria data in Shandong Province from 2005 to 2009 (control phase) and from 2010 to 2015 (elimination phase) via web-based infectious diseases information management reporting system were reviewed and analysed [6–15]. Each case was required to distinguish indigenous case from imported case after the “1-3-7” strategy (which refers respectively to reporting of malaria cases within 1 day, case confirmation and investigation within 3 days, and foci investigation and response to prevent further transmission within 7 days) launched in 2012 [16]. The clinical diagnosed case was defined as patients with malaria-like symptoms but no parasite detected upon blood examination, while laboratory-confirmed case was defined as case confirmed by using any of the following methods including microscopy, rapid diagnostic tests (RDTs) or polymerase chain reaction (PCR).

In China, case was defined as indigenous refer to malaria acquired by mosquito transmission in an area where malaria is prevalent within China. Imported malaria case must meet all of the following criteria: by giving a diagnosis of malaria and with a travel history to malaria transmission season; and the onset time of the patient being less than 1 month after returning to China during the local transmission season. Both types of cases were included in this study. Population data for every county in Shandong from 2005 to 2015 were obtained from the National Bureau of Statistics of China.

Month index was used to understand month patterns of malaria incidence. The index was calculated as the case number for a given month (i.e. January) divided by the mean of case number in that corresponding month (i.e. January) during the timeframe of 2005–2015. No obvious month pattern was expected if the month index of each month was close to 1.0.

### Study site

The study was conducted in Shandong Province, one of the 31 provinces, located in eastern China. It has a long coastline of 3024.4 km and 296 islands. This province has 97.9 million population and 212 malaria cases in 2015, all are imported cases and account for 6.8% of total malaria cases in China in this year. The total number of Chinese labourers abroad was 59,941 in 2014 in Shandong, and this was increased by 57.7%, from the 2005 figure.

Shandong Province could be divided into four parts: Eastern Lu (Shandong was abbreviated as Lu in Chinese words), Southern Lu, Central Lu and Northern Lu. The Eastern Lu, Southern Lu, Central Lu and Northwest Lu harbored 32, 39, 51 and 15 counties, respectively. For Eastern Lu, the area of where closed to the Bohai Sea and Yellow Sea, the overseas trades were developed, therefore, the imported cases were predominant in this region. For Southern and Central Lu, these regions were border with Jiangsu Province and Henan Province, and historically was a severe endemic area. Northwest Lu was adjacent to Hebei Province and was regarded as a non-endemic region.

### Data analysis

Spatial cluster analysis was performed to test whether the malaria cases were distributed randomly over space and, if not, to evaluate any identified spatial disease clusters for statistical significance. SaTScan software (version 9.4.3, MA 02215, USA) was used to implement the spatial cluster analysis as described before [17, 18]. The SaTScan software tested for possible clusters within the variable window around the centroid of each county group. Cluster analysis was performed with the default maximum spatial cluster size of <50% of the population and use “Retrospective Space–Time” to analyse the distribution of indigenous and imported malaria cases in Shandong from 2005 to 2015 for control and elimination phase. The relative risk (RR) of malaria was tested by spatial scan between any county groups. All malaria cases reported in 2005–2015 were geo-coded and matched to the county-level layers of polygon and point using the software ArcGIS 10.1(ESRI Inc., Redlands, CA, USA). *p* values <0.05 were considered to be statistically significant. Annual parasitic incidence (API) = total confirmed cases in a year × 1000/total population.

## Results

### Epidemiological study in Shandong Province, 2005**–**2015

From 2005 to 2015, Shandong Province had reported 1564 malaria cases and 27.1% of them were indigenous (n = 424). In the control phase (2005**–**2010), the local cases reached to peak in 2007 (n = 104) with the highest incidence (0.019/10,000) (Table 2). To solve this, Shandong had adopted the integrated control and elimination strategy and the cases was declined to the lowest level in 2012 (n = 93) and after then exhibited an increasing trend due to the imported cases. The infection source of each case and the laboratory result was clearly clarified after 2012 (Table 2).

**Table 2:** Malaria trend in Shandong Province, 2005–2015

Year	Total	API	Indigenous	*P. f*	*P. v*	*P. m*	*P. o*	Unclassified	Death	Laboratory diagnosed	Male patients (%)
2005	126	0.014	52	6	95	0	0	25	1	75	77.0
2006	149	0.016	90	8	129	0	0	12	0	108	72.5
2007	176	0.019	104	16	160	0	0	0	1	127	72.2
2008	174	0.019	72	40	114	0	0	20	5	115	82.2
2009	120	0.013	50	23	74	0	0	23	0	84	86.7
2010	117	0.013	37	40	55	0	0	22	0	83	82.1
2011	116	0.013	19	73	38	0	0	5	3	101	89.7
2012	93	0.010	0	67	21	0	3	2	0	76	98.9
2013	131	0.014	0	116	7	4	4	0	0	131	97.7
2014	150	0.016	0	121	16	4	9	0	3	150	100.0
2015	212	0.022	0	164	21	9	18	0	2	212	99.5
Total	1564	–	424	674	730	17	34	109	15	1262	–

**Fig. 2:**
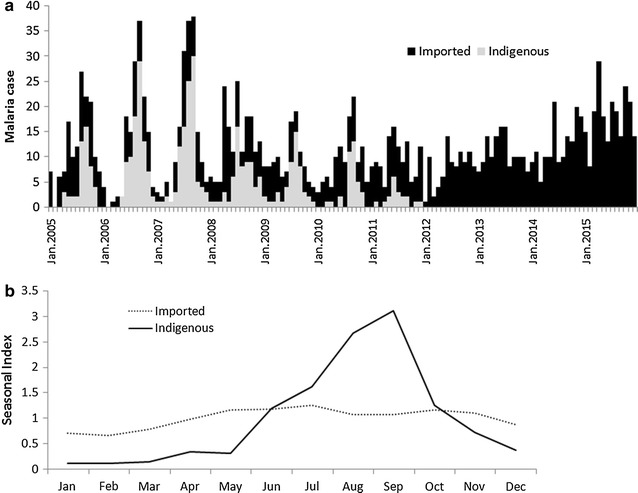
Malaria cases occurred in Shandong Province, 2005–2015. **a** The indigenous (*black*) and imported (*grey*) cases distribution and **b** month index of indigenous (*solid line*) and imported (*dash line*) cases

Shandong had eliminated local transmission in 2012, but the proportion of imported cases had begun to increase (Fig. 2a) [19, 20]. The factor determining this was the increasing imported *P. falciparum* coming back from African countries. Due to this, the reported *P. falciparum* with a 26-folds increase was noted from 2005 (n = 6) to 2015 (n = 164) in Shandong, in which the proportion of imported *P. falciparum* increased from 2005 (4.8%) to 2015 (77.4%). In 2015, a total of 212 malaria cases were reported, and 77.4% of them were identified as *P. falciparum*. In addition, the imported malaria was spread to most cities of Shandong, including Jining (n = 214 [13.7%]), Weihai (n = 151 [9.7%]), and Yantai (n = 107 [6.8%]).

The temporal distribution of malaria cases varied based on the infection source. As for the indigenous cases, most of them occurred in June-October (n = 339, 80.0%, 339/424), while no significant seasonal difference was observed for imported cases (χ^2^ = 32.546, *p* > 0.05); cases were mainly reported during May to November (n = 766, 67.2%, 766/1140), but this was because of workers returning to China from other countries to perform agricultural work during this period (Fig. 2b).

Among all the cases, *P. vivax* and *P. falciparum* were the predominant species (n = 730, 46.7% and 674, 43.1%, respectively). *Plasmodium ovale* and *Plasmodium malariae* were firstly reported in 2012 and 2013, from patients returned from Equatorial Guinea and Angola, respectively [21]. The occurrence of imported *P. ovale* and *P. malariae* cases has been increasing in this region, as in the entire county.

The malaria cases occurred 87.0% in male and 13.0% in female with similar distribution for all age groups. The highest malaria cases were observed in the age group of 36–40, 89.0% in male and 11.0% in female.

Shandong had reported 15 death cases caused by severe *P. falciparum* malaria, among which one third cases occurred in 2008. The death patients, aged from 26 to 64, had returned from African countries, and the average interval from onset to diagnosis was 9 days (ranging from 2 to 26 days).

### Type II and Type III counties

**Fig. 3:**
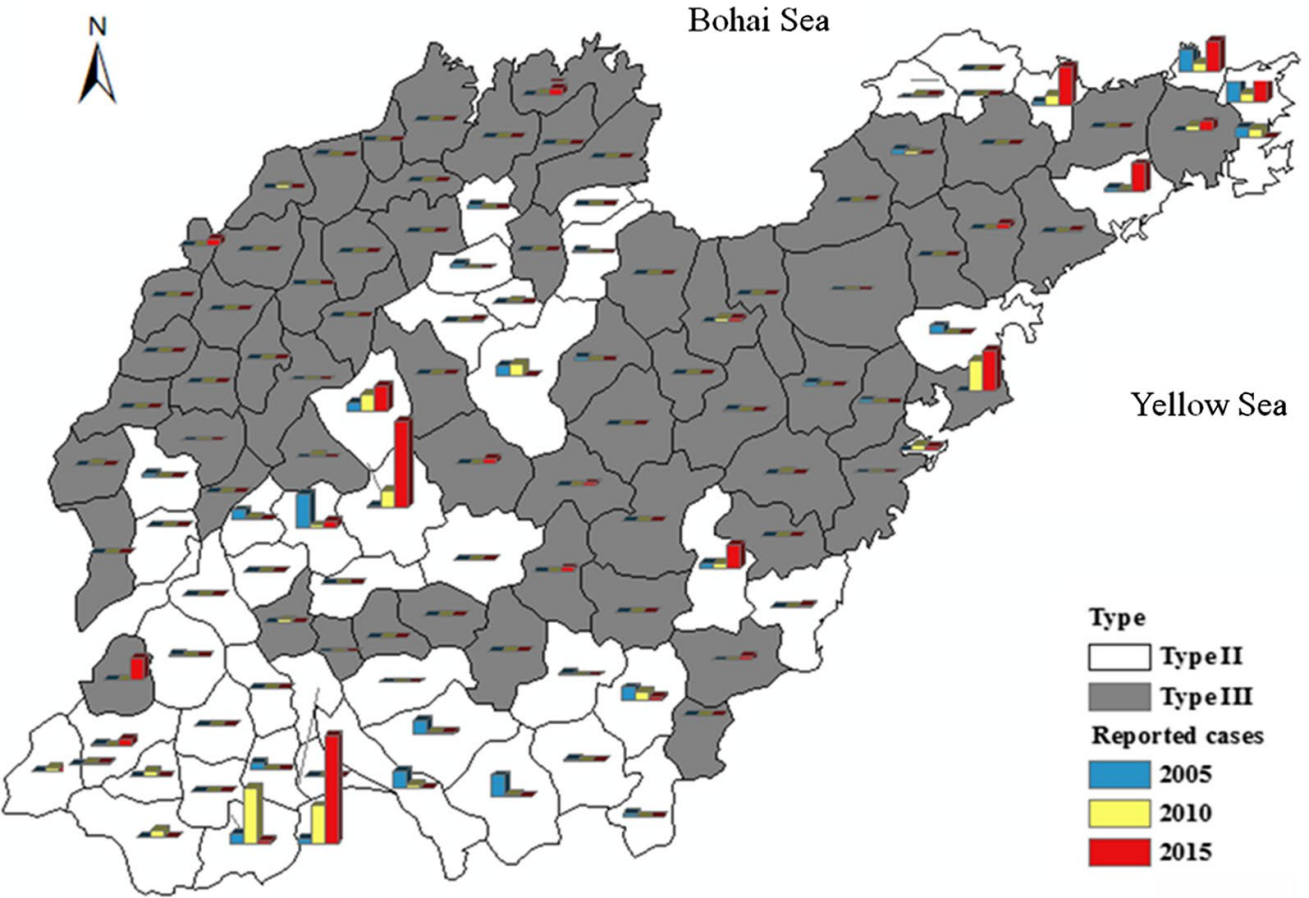
Malaria in Type II and Type III counties in Shandong Province, 2005–2015. The type of counties were represent using color of *white* (Type II) and *black* (Type III). The reported cases in 2005 (*blue*), 2010 (*yellow*) and 2015 (*red*) were indicated in each counties in the map

Shandong Province has 137 counties, which were classified into 58 Type II counties and 79 Type III counties according to NMEAP. Most of the cases (n = 1247, 79.7%) in Shandong occurred in the Type II counties. In 2005, there are 31 Type II counties and 11 Type III counties, reporting 110 and 16 cases, respectively, and mainly from the South and Central Lu (Fig. 3). In 2015, a total of 34 counties, including 20 Type II and 14 Type III, reported 161 and 51 cases, respectively (Fig. 3). The scale of Type II counties reported malaria cases decreased (from 31 to 20), while the number of Type III counties reported cases remained basically unchanged in the same timeframe.

### Regional distribution and spatial cluster analysis

**Fig. 4:**
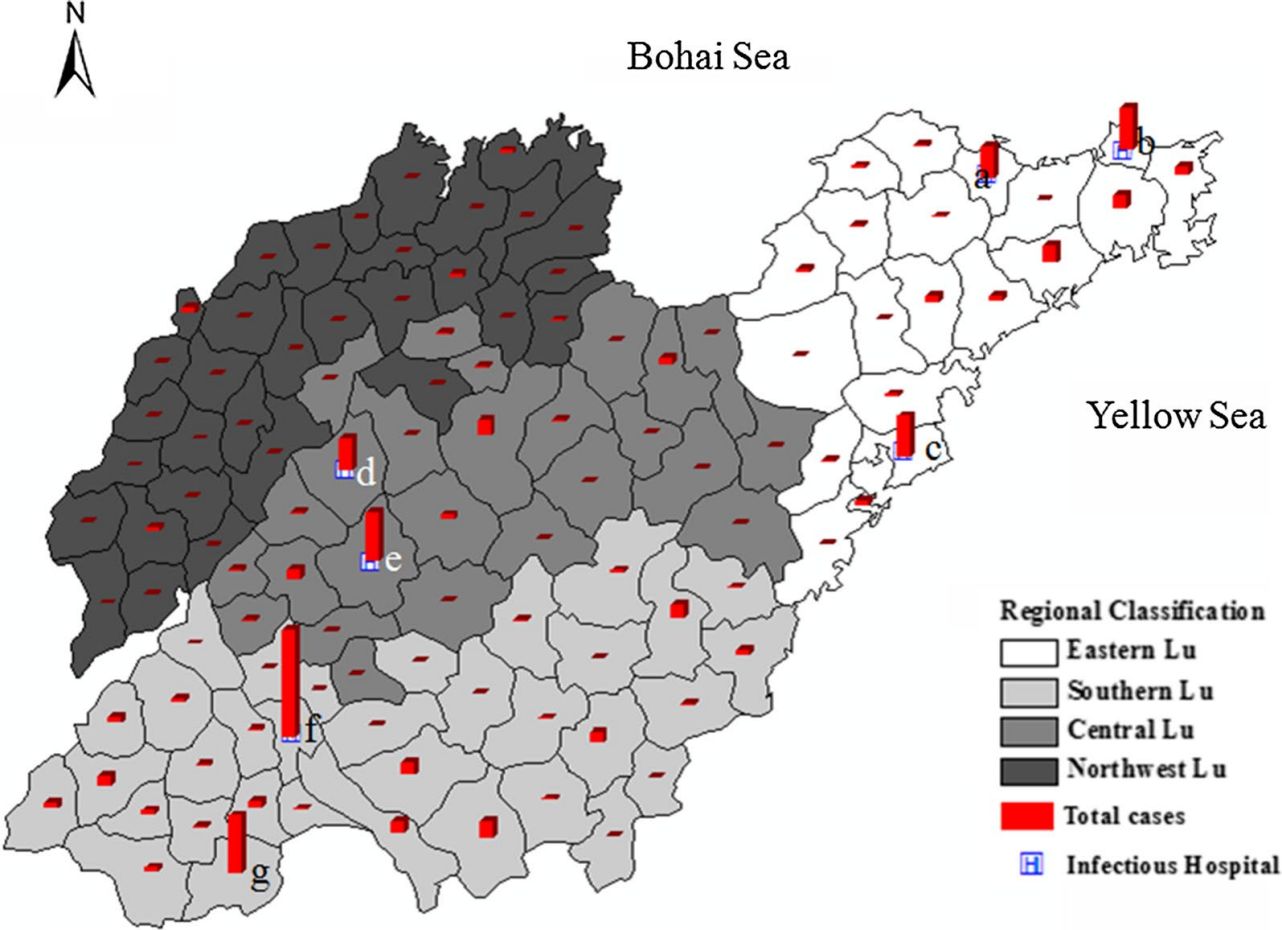
Regional classification of malaria in Shandong Province, 2005–2015. The *white*, *grey* and *black* represent the Eastern Lu, Southern Lu and Northern Lu, respectively. The *red* column indicated the total malaria cases reported in 2005–2015 in each county. The *square H* represents the infectious hospitals set up in Shandong Province. The *letter* in the map was indicated as following: *a* Zhifu, *b* Huancui, *c* Shibei, *d* Shizhong, *e* Taishan, *f* Rencheng, *g* Shanxian

From 2005 to 2015, Eastern Lu, Southern Lu, Central Lu and Northwest Lu reported 424, 710, 377 and 53 malaria cases, respectively. Most of the cases (n = 795, 50.8%) occurred in the counties of Rencheng (n = 238, 15.2%), Shanxian (n = 128, 8.2%), Taishan (n = 107, 6.8%), Huancui (n = 93, 5.9%), Shibei (n = 91, 5.8%), Zhifu (n = 69, 4.4%) and Shizhong (n = 69, 4.4%). Each of these counties has an infectious diseases hospital, except for Shan County, and these hospitals have adequate technical support, as well as RDTs and anti-malarials, and have the capability of carrying out diagnosis and treatment (Fig. 4). In addition, the indigenous cases (80.0%, 339/424) and *P. vivax* cases (57.5%, 420/730) occurred mainly in Southern Lu.

**Fig. 5:**
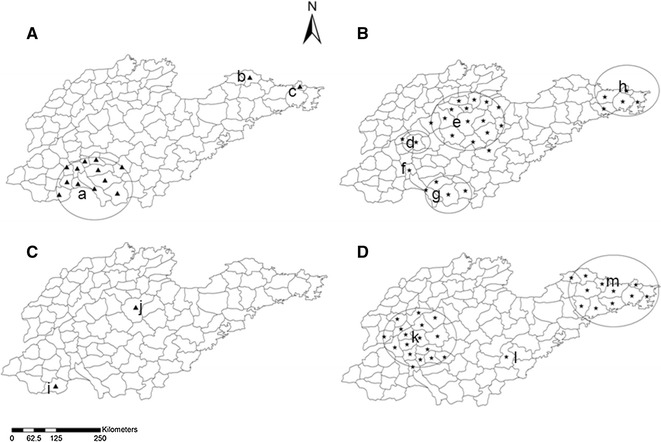
Proposed cluster areas in Shandong Province, 2005–2015. The time period was investigated for control phase (2005–2009) and elimination phase (2010–2015). **A** Indigenous case in 2005–2009 containing *a*–*c*, **B** imported case in 2005–2009 containing *d*–*h*, **C** indigenous case in 2010–2015 containing *i* and *j*, and **D** imported case in 2010–2015 containing *k*–*m*. The cluster analysis was performed with the default maximum spatial cluster size of <50% of the population

Regarding to the spatial cluster analysis, using the maximum spatial cluster size of <50% of the total population, the spatial cluster analysis indicated that the both indigenous and imported cases, in control phase (2005–2009) and elimination phase (2010–2015), were not distributed randomly. For the indigenous cases in control phase, three proposed clusters were identified with 126, 5 and 6 cases, respectively (Fig. 5a), while five proposed clusters were identified for imported cases during the same timeframe (Fig. 5b). For the indigenous cases in elimination phase, only two proposed clusters named Shan County and Zhangdian County with 37 and 4 cases, respectively (Fig. 5c), in contrast, the imported cases were clustered in three areas with 236, 113 and 17 cases, which was 48.0% of the total cases in the elimination phase (Fig. 5d). All the proposed clusters with relative risk level were indicated in Table 3.

**Table 3:** Spatial cluster analysis in Shandong Province, 2005–2015

Phase	Infection source	Code	No. of counties	Proposed cluster timeframe	No. of cases	Expected cases	Population (10,000)	RR	p
Control	Indigenous	a	13	Jan. 1 of 2006 to Dec. 31 of 2007	126	18.80	2165.7	9.78	<0.001
		b	1	Jan. 1 of 2008 to Dec. 31 of 2008	5	0.20	44.9	25.90	0.013
		c	1	Jan. 1 of 2005 to Dec. 31 of 2005	6	1.10	254.3	5.51	0.698
	Imported	d	2	Jan. 1 of 2005 to Dec. 31 of 2005	15	0.62	133.0	25.17	<0.001
		e	14	Jan. 1 of 2005 to Dec. 31 of 2005	20	9.29	1994.4	2.22	0.856
		f	1	Jan. 1 of 2008 to Dec. 31 of 2009	28	7.55	809.9	3.92	<0.001
		g	4	Jan. 1 of 2007 to Dec. 31 of 2007	15	3.91	840.2	3.95	0.037
		h	5	Jan. 1 of 2008 to Dec. 31 of 2009	45	4.49	481.1	11.23	<0.001
Elimination	Indigenous	i	1	Jan. 1 of 2010 to Dec. 31 of 2011	37	0.14	120.2	801.58	<0.001
		j	1	Jan. 1 of 2010 to Dec. 31 of 2011	4	0.46	411.7	9.19	0.732
	Imported	k	17	Jan. 1 of 2013 to Dec. 31 of 2015	236	68.53	2970.6	4.54	<0.001
		l	1	Jan. 1 of 2013 to Dec. 31 of 2015	17	2.48	107.5	6.99	<0.001
		m	11	Jan. 1 of 2013 to Dec. 31 of 2015	113	33.54	1453.7	3.78	<0.001

## Discussion

This study demonstrated the great changes occurring refer to the epidemiological characteristics of malaria in Shandong Province during the period transition from intensified control to elimination. Three epidemiological characteristics were observed: Firstly, the indigenous case was sharply declined like the whole country, while imported cases was increased dramatically due to the large number of returned migrate workers from African and Southeast Asia countries.

Shandong had experienced a malaria transmission peak during 2006–2009, which was mainly caused by the reemergence of malaria in the central China along the Huai River covering Anhui, Henan, Hubei, Jiangsu and Shandong provinces. The residual foci, changing meteorological factors, and increasing mobile population may contributes to the reemergence in these regions [22]. This was seen herein that, during the control phase, the indigenous cases were clustered in the Southern Lu, neighbouring with Jiangsu Province. For example, in 2006–2009, 255 indigenous cases in Southern Lu represented 69.3% of all indigenous cases in Shandong. After substantial efforts were undertaken to strengthen malaria control at all levels, as well as the support of the Global Fund Rounds Five and Six, the epidemics have been well controlled. The intensified anti-malarial measures, including active case detection, case management, vector control interventions (with insecticide-treated nets distribution, indoor residual spraying and environment improvement), radical treatment that administration with primaquine, and prophylactic chemotherapy with pyrimethamine in high-transmission settings [23, 24]. In addition, Shandong also adopted mobile population management plan from 1985, particularly of migrant workers, ex-servicemen, students from endemic areas such as Hainan and Yunnan Provinces [25]. In this study, the imported cases cluster region during control phase were mainly domestically from Yunnan, Hainan and other endemic area. For example, in 2008, 75 malaria cases (43.1%, 75/174) were domestically-mobile cases, most of them (69.3%) came back from Yunnan Province [26]. In order to control epidemic in the high-risk setting, Shandong had set up malaria posts and screens febrile patients for malaria by microscopy. Since 1980s, more than 900 posts were established covering 20 million population in 38 counties of Southern Lu, focus on the suspected malaria and febrile patients. Nearly 100,000 population were screened each year and this timely discovered infection source and providing great access for the corresponding treatment [25].

Secondly, the proportion of *P. falciparum* cases increased while the proportion of *P. vivax* cases decreased, which could be explained by an increase of imported cases from patients having returned from Africa.

After NMEAP was launched in 2010, imported cases have become the greatest challenge and are clustered in two regions [27], Eastern Lu and Central Lu. To deal with this, Shandong has reshaped the surveillance system to establish “provincial-municipal-county-township” network to rapidly and timely detect every case and ensure each case gets prompt and appropriate diagnosis and treatment. The “1-3-7” strategy, launched in 2012, is a simplified set of targets that delineates responsibilities and actions with the indicators as following: 1, case reporting within 1 day; 3, case investigation within 3 days and 7, focus investigation and response within 7 days [16]. The “1” indicator was accessible because of the widespread use of mobile phones and internet throughout. Efforts are being made to deal with indicators “3” and “7”, by reducing the time required to transport and process samples. Another strategy support was the establishment of the “provincial-municipal-county” network reference laboratory, a network focusing on case diagnosis and verification, establishment of a quality assurance sample bank, capacity training and technical supports at different levels in order to ensure accuracy and reliability of diagnostic results [28]. Shandong has adopted different control and prevention strategies in different endemic areas. For example, for Eastern Lu and Central Lu where migration is frequent, the staff of the Centres for Disease Control and Prevention (CDC) carry out health education mainly to the overseas labourers, who usually work on construction sites, with poor living conditions and lack of access to mosquito control measures [29]. Most Chinese workers lack immunity to the local *Plasmodium* species, combined with poor awareness of personal protection and misuse of prophylactic anti-malarial drugs [30, 31]. In recent years, there has been an increase of *P. malariae* and *P. ovale* cases, which had previously been absent from Shandong. Chloroquine and primaquine are given for 8 days to treat *P. ovale* cases, as for *P. vivax* [32]. These *P. ovale* cases were all re-confirmed by PCR in reference laboratories in the provincial CDC, as it is often difficult to distinguish the two species by microscopy. Finally, Shandong has set up a strong multisectoral collaboration and cooperation with organizations, such as CDC, Entry-Exit Inspection and Quarantine Bureau, Public Security Bureau, and Education Bureau. Shandong also participate in the “Joint Malaria Control and Prevention in Central Five Provinces” programme, launched in 1974 and carry out the supervision and inspection each year, which further strengthens communication and experience sharing between the provinces and facilitates the malaria control process [33].

Thirdly, the disease clustered in some regions with infectious diseases hospitals, such as Jinning, Jinnan, Qingdao and Taian. This was because the returned migrant workers are given the health education to seek diagnosis and treatment in infectious hospitals for free once the symptom occurred.

This study addresses the important topic of maintaining malaria elimination efforts in Shandong and attempts to use surveillance records to identify trends and spatial patterns. This decade coincided with intensive malaria control and elimination efforts as well as increased migration. With zero indigenous malaria, prompt case detection and treatment by health care providers is a major challenge, which is a similar process to achieve the malaria elimination as other countries [34–36]. To achieve the elimination in Shandong as scheduled, proactive mapping of disease transmission hotspots and the genetic diversity and population dynamics will be effective means of facilitating early detection and interruption of the remaining transmissions [37–39].

## Conclusions

As the rest of the country, Shandong faces an increasing importation of malaria by returning migrants from Africa and Southeast Asia, and this may become catastrophic if no preventive efforts are undertaken. For prompt diagnosis and treatment with increasing imported cases in Shandong, the “1-3-7” elimination strategy should be carried out and all the indicators and timeframe are required to ensure elimination of residual potential reservoirs and prevention of indigenous cases outbreak by imported pathogens. The government should continue to maintain the emphasis on the malaria elimination process and guarantee funding. The effective multisectoral cooperation and coordination mechanisms should be strengthened. Capacity building should also be improved mainly focus on the surveillance, emergence response and case diagnosis and treatment. Furthermore, during the elimination phase, scientific research and cooperation with universities, research agencies and institutions should be established and strengthened.
